# Supplementary material for: Efficacy of signal peptide predictors in identifying signal peptides in the experimental secretome of Picrophilous torridus, a thermoacidophilic archaeon
Source: PLoS One. 2021 Aug 6;16(8):e0255826. doi: 10.1371/journal.pone.0255826 (PMC8345856; doi:10.1371/journal.pone.0255826)
Supplement: S1 Table — (DOCX) [file pone.0255826.s002.docx]

**S1 Table.** ***P.torridus* secretory proteins, PDB BLAST hits with percentage identity**

| **S. No.** | **Protein Accession** | **PDB BLAST hit ( % identity)** |
| --- | --- | --- |
|  | Q6KZA7 | 6N2N_A (34.23) |
|  | Q6KZE9 | 2R7A_A (21.21) |
|  | Q6KZF2 | 1BVU_A (61.40) |
|  | Q6KZS2 | 1A6D_B (79.31) |
|  | Q6L081 | 3ZKK_A (21.21) |
|  | Q6L0B7 | 5B46_A (45.05) |
|  | Q6L0M9 | 2RIF_A (29.63) |
|  | Q6L0W3 | 1PMA_A (77.68) |
|  | Q6L0Y1 | 3VGP_A (28.75) |
|  | Q6L140 | 5OVQ_A (50.71) |
|  | Q6L182 | 4OET_A (22.85) |
|  | Q6L1B1 | 6SKF_BG (46.10) |
|  | Q6L1T2 | 3RCY_A (34.18) |
|  | Q6L202 | 1JNY_A (58.27) |
|  | Q6L248 | 1J08_A (37.28) |
|  | Q6L2C8 | 5A8J_A (32.25) |
|  | Q6L2M0 | 6L8A_A (29.61) |
|  | Q6L2N0 | 1ZVK_A (32.42) |
