# Supplementary material for: Efficacy of signal peptide predictors in identifying signal peptides in the experimental secretome of Picrophilous torridus, a thermoacidophilic archaeon
Source: PLoS One. 2021 Aug 6;16(8):e0255826. doi: 10.1371/journal.pone.0255826 (PMC8345856; doi:10.1371/journal.pone.0255826)
Supplement: S2 Table — (DOCX) [file pone.0255826.s003.docx]

**S2 Table.** Prediction efficacy of various SPPs in identifying SPs in *P. torridus* secretome identified in three independent experiments

| **Dataset (total number of proteins in the secretome)** | **PRED-SIGNAL (prediction efficacy in percentage)** | **SignalP 5.0**  **(prediction efficacy in percentage)** | **PRED-TAT(prediction efficacy in percentage)** | **LipoP 1.0**  **(prediction efficacy in percentage)** |
| --- | --- | --- | --- | --- |
| S1 File (68) | 12 (17.64%) | 12 (17.64%) | 13 (19.11%) | 11 (16.17%) |
| S2 File (76) | 12 (15.78%) | 10 (13.15%) | 11(14.47%) | 10 (13.15%) |
| S3File (97) | 15 (15.46%) | 14 (14.43%) | 16 (16.49%) | 11(11.34%) |
| Mean prediction efficacy) | 16.29 % | 15.07% | 16.69 % | 13.55% |
